# Single Mothers by Choice: Mother–Child Relationships and Children’s Psychological Adjustment

**DOI:** 10.1037/fam0000188

**Published:** 2016-02-11

**Authors:** Susan Golombok, Sophie Zadeh, Susan Imrie, Venessa Smith, Tabitha Freeman

**Affiliations:** 1 Centre for Family Research, University of Cambridge; 2 London Women’s Clinic, London, UK; 3 Centre for Family Research, University of Cambridge

**Keywords:** solo mothers, single mothers by choice, parenting, child adjustment

## Abstract

Fifty-one solo mother families were compared with 52 two-parent families all with a 4–9-year-old child conceived by donor insemination. Standardized interview, observational and questionnaire measures of maternal wellbeing, mother–child relationships and child adjustment were administered to mothers, children and teachers. There were no differences in parenting quality between family types apart from lower mother–child conflict in solo mother families. Neither were there differences in child adjustment. Perceived financial difficulties, child’s gender, and parenting stress were associated with children’s adjustment problems in both family types. The findings suggest that solo motherhood, in itself, does not result in psychological problems for children.

In recent decades, there has been a dramatic increase in the number of single-parent families. In both the United States (U.S. Census Bureau, 2012) and the United Kingdom (Lloyd & Lacey, 2012), 30% of households with children are headed by single parents, the large majority of whom are single mothers. These figures compare with less than 10% at the beginning of the 1970s. Single-mother families are formed in a number of ways. Parental divorce or separation is the most common reason for children to be raised in single-mother families. There has also been a rise in the number of children born to single unmarried mothers as a result of unplanned pregnancies. However, the newest type of single-mother family comprises single heterosexual women who have chosen to parent alone and have had children through donor insemination. These women are generally referred to as “single mothers by choice” or “solo mothers” (Bock, 2000; Hertz, 2006; Weinraub, Horvath, & Gringlas, 2002) and these terms are used interchangeably below. The number of such families has risen sharply since the millennium and is likely to grow given the demographic shift toward older first-time motherhood (Graham, 2012). Indeed, a significant proportion of those who now seek fertility treatment with donated gametes are women without a male partner (De Wert et al., 2014).

Studies have shown that single mothers by choice are generally well-educated women in professional occupations who become mothers in their late 30s or early 40s (Bock, 2000; Graham, 2014; Graham & Braverman, 2012; Jadva, Badger, Morrissette, & Golombok, 2009; Murray & Golombok, 2005a; Weinraub et al., 2002). In spite of having chosen to parent alone, the majority of solo mothers do so not from choice, but because they do not have a current partner and feel that time is running out for them to have a child (Graham & Braverman, 2012; Hertz, 2006; Jadva et al., 2009; Murray & Golombok, 2005a). Many single mothers by choice report that they would have preferred to have children within a traditional family setting but could not wait any longer because of their increasing age and associated fertility decline. As Graham (2014) pointed out, if they wanted to become mothers they did not actually have a choice.

There is a large body of research on the psychological wellbeing of children in single-mother families formed by divorce. These studies have consistently shown that children whose parents divorce are more likely to show emotional and behavioral problems than are children in intact families (Amato, 2000, 2001, 2005; Coleman & Glenn, 2009; Hetherington & Stanley-Hagan, 1999; Pryor & Rodgers, 2001). However, the children’s difficulties appear to be largely associated with aspects of the divorce, rather than single-parenthood, in itself. One factor that has been found to be related to children’s adjustment problems is conflict between parents (Amato, 2000, 2005; Pryor & Rodgers, 2001). The financial hardship that is often experienced by single-parent families following divorce has also been shown to be associated with children’s psychological problems (Amato, 2000, 2005; Hetherington & Stanley-Hagan, 2002; McLanahan & Sandefur, 1994; Pryor & Rodgers, 2001). Furthermore, a number of studies have demonstrated a link between parental depression, poor parenting quality and negative child outcomes in single-parent families following divorce (Amato, 2000; Dunn et al., 1998; Hetherington & Stanley-Hagan, 2002).

There is also a growing research literature on children raised by unmarried single mothers. The Fragile Families Study in the United States found more negative mental health outcomes for children born to single unmarried mothers than to married parents, even after differences in parental resources had been controlled for (Waldfogel, Craigie, & Brooks-Gunn, 2010). As with single-mother families formed by divorce, economic disadvantage, parental mental health problems, and poor parenting quality were associated with more negative outcomes for these children. Similar findings were reported from the Millennium Cohort Study in the United Kingdom (Kiernan & Mensah, 2010). Children born to single mothers showed high rates of psychological problems associated with high levels of economic disadvantage and poor maternal mental health, with raised levels of behavioral problems still apparent after taking account of maternal depression and socioeconomic status.

Unlike divorced or unmarried single mothers who have had unplanned pregnancies, single mothers by choice make an active decision to parent alone, and thus differ from those who unintentionally find themselves in this situation. Children of single mothers by choice have not been exposed to parental conflict and are less likely to have experienced the economic hardship or maternal psychological problems that commonly result from marital breakdown and unplanned single parenthood (Hertz, 2006; Jadva et al., 2009; Murray & Golombok, 2005a). Nevertheless, they grow up without a father from the start and, for those conceived by donor insemination at a fertility clinic, do not know the identity of their biological father. Even in countries where the use of anonymous donors is prohibited, children are not able to discover his identity until late adolescence. This makes them distinct from most other children of single mothers, whose fathers may be absent but whose identity is known.

There is little research on the development and well-being of children born to single mothers by choice. In a comparison between 27 single heterosexual mother families and 50 married heterosexual parent families, all with infants conceived by donor insemination, no differences were identified between the two family types in terms of mothers’ psychological well-being, adaptation to motherhood, expressed warmth, and emotional involvement or bonding with their infants (Murray & Golombok, 2005a). However, the single mothers showed lower levels of interaction and sensitive responding to their infants than did the married mothers, possibly because the presence of a partner allowed the married mothers more time with their babies. The families continued to function well as the children reached 2 years old (Murray & Golombok, 2005b). Although mothers from both types of family showed positive relationships with their children, the single mothers showed greater joy and less anger toward their children as assessed by the Parent Development Interview, an interview technique designed to assess the nature of the emotional bond between the mother and the child (Slade, Belsky, Aber, & Phelps, 1999). With respect to the children, those with single mothers showed fewer emotional and behavioral problems than did those with married mothers. However, at age 2, the children of single mothers were too young to understand the social significance of the absence of a father.

The only controlled study of older children focused primarily on lesbian mother families (Chan, Raboy, & Patterson, 1998). Comparisons between 30 solo mother families and 50 two-parent families with 7-year-old children conceived by donor insemination found no differences in conduct or emotional problems, social competence, or adaptive functioning between the children of single and partnered mothers. However, no direct comparisons were conducted between the nine heterosexual solo mothers and the 16 heterosexual partnered mothers in the sample.

The aim of the present investigation was to add to the small but growing body of research on solo mother families by conducting an in-depth, multimethod, multi-informant, controlled study of families with children who were old enough to understand that they did not have a father. Developmental contextual systems theory (Overton, 2014, 2015) provided the underlying conceptual framework. In accordance with that theory and findings obtained in previous research, it was hypothesized that children’s adjustment would not be a direct function of the number of parents in the family but would instead be associated with the quality of mother–child relationships, with both the quality of those relationships and the children’s adjustment being directly and indirectly affected by indices of the families’ financial difficulties and maternal mental health problems. The other key risk factor for single-mother families, parental conflict, is not applicable to single mothers by choice.

## Method

### Participants

Fifty-one heterosexual single mothers by choice (solo mothers) and a comparison group of 52 heterosexual married or cohabiting mothers participated in the study. The children in both groups of families were conceived by donor insemination to control for the use of third-party assisted reproduction in the birth of the child. The families were recruited through the London Women’s Clinic, one of the largest fertility clinics in the United Kingdom that has also provided the longest-standing program for single women (Human Fertilisation and Embryology Authority, 2013). Single-mother and two-parent families with a donor-conceived child aged between 4 and 9 years were invited to take part in the study. The inclusion criteria for the solo mothers were that they had not cohabited since the birth of the child, had not been involved in a noncohabiting relationship for longer than 6 months, and had not used egg donation in addition to donor insemination to conceive their child. Partnered mothers were required to be still living with the child’s father. Solo mothers and partnered mothers who met the inclusion criteria were asked by the clinic to take part in the research. A random sample of solo mothers was selected by the clinic, representing around 70% of those mothers who met the study criteria, and the partnered mothers matched on the age band and gender of the child. A participation rate of 72% was obtained.

As shown in Table 1, there was no significant difference between family types in the age of the target child, *F*(1, 101) = 1.79, *p = ns*, with the average age being 66 months. Neither was there a significant difference with respect to the children’s gender, χ^2^(1) = 0.77, *p* = *ns*. All of the children attended school or nursery/preschool. The age of the mother did differ significantly between family types, *F*(1, 101) = 36.05, *p* < .001, reflecting the older age of the solo mothers (mean age 44 years) than the partnered mothers (mean age 39 years). There was also a significant difference between family types in the number of siblings in the family, χ^2^(2) = 9.39, *p* < .01, with children in solo mother families having fewer siblings. The mothers in the two family types did not differ in educational level, χ^2^(1) = 1.87, *p* = *ns*; perceived financial difficulties, χ^2^ (2) = 0.32, *p = ns*; or treatment for psychiatric problems in the previous year, χ^2^(2) = 3.08, *p = ns.* However, they did differ in working status, χ^2^(2) = 8.54, *p* < .05, reflecting a higher proportion of solo mothers than partnered mothers in full-time employment. All of the mothers were White with the exception of three Asian mothers (two solo mothers and one partnered mother) and three Black mothers (two solo mothers and one partnered mother). All of the fathers in two-parent families were involved in caring for the child.[Table-anchor tbl1]

### Procedure

The families were assessed at home. Written informed consent to participate in the investigation was obtained from each parent and verbal assent was obtained from the child. Ethical approval was granted by the University of Cambridge Psychology Research Ethics Committee. Each parent was administered an audiorecorded standardized interview that lasted approximately 1.5 hr and standardized questionnaires and participated in a video-recorded observational task with the child that lasted 5–10 min. Teachers completed a questionnaire designed to assess the children’s psychological adjustment. Written informed consent was obtained from teachers. To provide interrater reliability ratings for the interview and observational measures, data from 30 randomly selected families were coded by a second interviewer who was blind to family type.

### Measures

#### Parenting

##### Parenting interview

The mothers were interviewed using an adaptation of a semistructured interview designed to assess quality of parenting that has been validated against observational ratings of mother–child relationships in the home (Quinton & Rutter, 1988) and has been used successfully in previous studies of donor conception families with children of the same age (Golombok et al., 2011). Detailed accounts are obtained of the child’s behavior and the parent’s response to it, with particular reference to interactions relating to warmth and control. A flexible style of questioning is used to elicit sufficient information for each variable to be rated by the researcher using a standardized coding scheme based upon a detailed coding manual. Thus ratings are carried out by the researcher using in-depth information obtained from the mother rather than by the mother herself.

The following variables were coded: (a) expressed warmth from 0 (*none*) to 5 (*high*) took account of the mother’s tone of voice, facial expressions and gestures in addition to what the mother said about the child; (b) mother-to-child warmth from 0 (*little or none*) to 3 (*high*) represented the frequency and spontaneity of affection shown by the mother to the child; (x) child-to-mother warmth from 0 (*little or none*) to 3 (*high*) represented the frequency and spontaneity of affection shown by the child to the mother; (d) mother’s enjoyment of play from 1 (*little or none*) to 4 (*a great deal*) assessed the extent to which the mother enjoyed playing with the child; (e) amount of interaction from 1 (*little*) to 3 (*high*) assessed the amount of time the mother and child spent in shared activities; (f) quality of interaction from 1 (*poor*) to 4 (*very good*) was based on the extent to which the mother and child wanted to be with each other and enjoyed each other’s company; (g) conflict from 0 (*little or none*) to 3 (*a great deal*) assessed the extent of disagreement between mothers and their children; (h) frequency of battles from 0 (*never/rarely*) to 8 (*few times daily*) assessed the frequency of mother–child conflict; (i) level of battles from 0 (*none*) to 3 (*major*) assessed the severity of mother–child conflict; and (j) criticism from 0 (*none*) to 4 (*considerable*) was based on the amount of criticism of the child by the mother. The interrater reliabilities were calculated using Cohen’s Kappa and transformed into interrater agreement rates using the thresholds provided by Bakeman and Quera (2011). Six variables showed agreement above 80% (mother-to-child warmth, mother’s enjoyment of play, amount of interaction, conflict, frequency of battles, and level of battles), two showed agreement above 70% (quality of interaction and criticism), and two showed agreement below 70% (expressed warmth and child-to-mother warmth).

##### Mothers’ psychological wellbeing

The Trait Anxiety Inventory (Spielberger, 1983), the Edinburgh Depression Scale (Thorpe, 1993), and the short form of the Parenting Stress Index (Abidin, 1990) were completed by the mothers to assess anxiety, depression and stress associated with parenting, respectively. Each of these instruments, for which higher scores represent greater difficulties, has been shown to have good reliability and to discriminate well between clinical and nonclinical groups.

##### Parent–child observations

The Etch-A-Sketch task (Stevenson-Hinde & Shouldice, 1995) was used to obtain an observational assessment of interaction between the mother and the child. The Etch-A-Sketch is a drawing tool with two dials that allow one person to draw vertically and the other to draw horizontally. The mother and child were asked to copy a picture of a house, each using one dial only, with clear instructions not to use the other dial. The sessions were video-recorded and coded using the Parent–Child Interaction System (Deater-Deckard & Petrill, 2004) to assess the construct of mutuality; that is, the extent to which the parent and child engaged in positive dyadic interaction characterized by warmth, mutual responsiveness, and cooperation. The following variables were rated on a 7-point scale ranging from 1 (*no instances*) to 7 (*constant, throughout interaction*): (a) child’s responsiveness to parent assessed the extent to which the child responded immediately and contingently to the mother’s comments, questions or behaviors; (b) mother’s responsiveness to child assessed the extent to which the mother responded immediately and contingently to the child’s comments, questions or behaviors; (c) dyadic reciprocity assessed the degree to which the dyad showed shared positive affect, eye contact and a “turn-taking” (conversation like) quality of interaction; and (d) dyadic cooperation assessed the degree of agreement about whether and how to proceed with the task. It was not possible to calculate Cohen’s Kappas for these variables due to restriction of the range of the scores as most families obtained scores at the top end of the scales. However, this did not reflect low interrater reliability as agreement within one point was 100% for dyadic reciprocity, above 95% for child’s responsiveness, above 90% for dyadic cooperation and above 85% for mother’s responsiveness.

#### Child Adjustment

##### Strengths and Difficulties Questionnaire

The presence of children’s emotional and behavioral difficulties was assessed with the Strengths and Difficulties Questionnaire (SDQ; Goodman, 1994, 1997) administered to the mother and the child’s teacher to produce total scores of child adjustment problems, with higher scores indicating greater problems. The SDQ has been shown to have good internal consistency, test–retest and interrater reliability, and concurrent and discriminative validity (Goodman, 1994, 1997, 2001). For example, based on an epidemiological sample of more than 10,000 children in the United Kingdom (Goodman, 2001), internal consistency (Cronbach’s alpha) was found to be 0.73, test–retest reliability after 4–6 months was 0.62, and, in terms of validity, scores above the 90th percentile predicted a substantially raised probability of independently diagnosed psychiatric disorders (mean odds ratio = 15.7). In a review of the reliability and validity of the SDQ based upon 48 studies involving more than 130,000 children, Stone, Otten, Engels, Vermulst, & Janssens (2010) found the psychometric properties of the SDQ to be strong.

##### Psychiatric ratings

The child’s psychological adjustment was also assessed during the interview with the mother using a standardized procedure (Rutter, Cox, Tupling, Berger, & Yule, 1975). Detailed descriptions were obtained of any emotional or behavioral problems shown by the child. These descriptions of actual behavior, which included information about where the behavior was shown, severity of the behavior, frequency, precipitants, and course of the behavior over the past year were transcribed and rated by a child psychiatrist who was unaware of the nature of the study. A high level of reliability (*r* = .85) between ratings made by social scientists and those made “blindly” by a child psychiatrist has been demonstrated for this procedure and validity has been established through a high level of agreement between interview ratings of children’s psychological problems and mothers’ assessments of whether or not their children had emotional or behavioral difficulties (Rutter et al., 1975). Psychological problems, when identified, were rated according to severity on a 3-point scale ranging from 0 (*no disorder*) through 1 (*slight disorder*) to 2 (*marked disorder*) and type (anxiety, conduct/oppositional disorder, mixed disorder, Autism Spectrum Disorder, ADHD and speech delay).

## Results

### Analysis Plan

In the first instance, a principal components analysis was conducted with the interview variables relating to parenting quality. Two factors, each with item loadings of at least 0.6, explained 50% of the variance. The first factor (comprising expressed warmth, mother-to-child warmth, child-to-mother warmth, mother’s enjoyment of play, amount of interaction, and quality of interaction) was labeled *positive parenting* and the second factor (comprising frequency of battles, level of battles, conflict and criticism) was labeled *negative parenting*. The correlation between the two factors was *r* = −.34, *p* < .001, showing a slight negative relationship between them.

Comparisons of parenting between the solo mother families and the two-parent families were conducted using multivariate analyses of covariance (MANCOVAs). These were carried out separately for positive parenting, negative parenting, mothers’ psychological wellbeing, and the observational assessment of mother–child interaction. Children’s psychological wellbeing was compared between family types using analyses of covariance (ANCOVAs). For each analysis, demographic variables that were significantly correlated with any of the dependent variables were entered as covariates. The analyses were conducted with and without the gender of the child as a between-subjects factor. As significant interactions between child gender and family type were found for the children’s measures only, the former analyses were reported for the other variables. To examine factors associated with child adjustment in both family types, hierarchical regression analysis was carried out.

### Parenting

As shown in Table 2, the positive parenting variables (expressed warmth, overt mother–child warmth, overt child-mother warmth, mother’s enjoyment of play, amount of interaction, and quality of interaction) were entered into a MANCOVA with family type (one-parent vs. two-parent) as the between-subjects factor and mother’s age, child’s age and mother’s working status as covariates. Wilks’ λ was not significant, *F*(6, 92) = 0.61, *p* = .72, showing that there was no difference in the level of positive parenting between the solo mother and two-parent families. When the negative parenting variables (frequency of battles, level of battles, conflict, and criticism) were entered into a MANCOVA with family type (one-parent vs. two-parent) as the between-subjects factor and child’s age, child’s gender, mother’s age, mother’s educational level, and perceived financial difficulties as covariates, Wilks’ λ was significant, *F*(4, 93) = 3.38, *p* = .01. One-way ANCOVAs identified a significant difference between groups for frequency of battles, *F*(1, 96) = 12.91, *p* = .001, *d*′ = 0.51, reflecting less frequent battles between mothers and children in solo mother than in two-parent families. When this analysis was repeated without covariates, there remained a significant difference in negative parenting between the two family types, *F*(4, 98) = 2.64, *p* = .04, again reflecting less frequent battles in solo mother than in two-parent families *F*(1, 101) = 6.67, *p* = .01. There were no significant differences between family types for level of battles, criticism, or conflict. When this analysis was repeated without covariates, there remained a significant difference in negative parenting between family types, again reflecting less frequent battles in single than in two-parent families.[Table-anchor tbl2]

In addition, a MANCOVA was carried out for the variables relating to the mothers’ psychological wellbeing (Trait Anxiety Inventory, Edinburgh Depression Scale, and Parenting Stress Index total scores) with family type (one-parent vs. two-parent) as the between-subjects factor and perceived financial difficulties as a covariate. Wilks’ λ was not significant, *F*(3, 91) = 0.56, *p* = .64, showing that there was no difference in parental wellbeing between the solo mother and two-parent families.

The variables relating to the construct of mutuality from the observational assessment of the quality of mother–child interaction (mother responsiveness, child responsiveness, dyadic reciprocity, and dyadic cooperation) were also entered into a MANCOVA with family type (one-parent vs. two-parent) as the between-subjects factor and mother’s age, and child’s age as covariates. Wilks’ λ was not significant, *F*(4, 81) = 1.05, *p* = .38, showing that there was no difference in mother–child interaction between the solo mother and two-parent families.

### Child Adjustment

A two-way ANCOVA with family type (one-parent vs. two-parent) and child’s gender (male vs. female) as between-subjects factors, and perceived financial difficulties entered as a covariate, was carried out for the total score of the Strengths and Difficulties Questionnaire (SDQ) rated by mothers. There was a significant main effect for child’s gender, *F*(1, 96) = 8.99, *p* = .003, showing higher levels of child adjustment problems among boys than girls. However, there was no significant difference between family types for the total score of the SDQ, *F*(1, 96) = 0.29, *p* = .59, and the interaction between family type and child’s gender was not significant, *F*(1, 96) = 0.61, *p* = .43.

Teachers’ total SDQ scores were also entered into a two-way ANCOVA with family type (one-parent vs. two-parent) and child’s gender (male vs. female) as between-subjects factors. The main effect for child’s gender was significant, *F*(1, 51) = 5.63, *p* < .05, indicating higher levels of child adjustment problems among boys than girls. Neither the main effect for family type, *F*(1, 54) = 1.23, *p* = .27, nor the interaction between family type and child’s gender, *F*(1, 54) = 1.70, *p* = .19, was significant. Although only 57% of the teachers completed the SDQ, the correlation between the mothers’ and teachers’ SDQ scores for those children for whom both questionnaires were available was significant (Pearson’s *r* = .53, *p* < 0.001). There was no significant difference in mothers’ total SDQ scores between those children for whom teachers’ SDQ scores were available and those for whom it was not, and no significant difference between family types in the proportion of teachers who did not complete this questionnaire.

The ratings of psychiatric disorder by a child psychiatrist found five children of solo mothers to show a slight disorder (one with speech delay, one with Autism Spectrum Disorder (ASD), one with conduct/oppositional defiant disorder (ODD), one with a mixed disorder including ADHD with ASD and ODD traits, and one with mixed disorder including ASD and speech delay) and two to show a marked disorder (one with speech delay and the other with a mixed disorder including speech delay, ADHD and anxiety). For the two-parent families, two children showed a slight disorder (one with ADHD and the other with speech delay) and two showed a marked disorder (one with speech delay and the other with a mixed disorder including ASD and speech delay). There was no difference in the proportion of children in solo mother and two-parent families rated as having a slight or marked psychiatric disorder, χ^2^(2) = 0.95, *p* = .62.

### Parenting and Child Adjustment

Hierarchical regression analysis was used to examine factors associated with child adjustment problems in both family types (see Table 3). The outcome variable was children’s SDQ scores as completed by mothers. The demographic variables that were significantly correlated with SDQ scores, perceived financial difficulties and child’s gender, were entered in the first step. The variables added in the second step were a dummy-coded variable representing family type as well as the parenting and mothers’ wellbeing variables from the interview (positive parenting, negative parenting, maternal depression [Edinburgh Depression Scale], maternal anxiety [Trait Anxiety Inventory], and maternal parenting stress [Parenting Stress Index]). In the third step, the interaction terms between family type and each of the five independent variables representing parenting and mothers’ wellbeing were included. Perceived financial difficulties and child’s gender jointly explained 13% of the variance in children’s adjustment and the omnibus test for the first step was significant: *F*(2, 92) = 6.83, *p* = .002, showing greater adjustment difficulties for children whose mothers were experiencing financial hardship and also for boys. The parenting and mothers’ psychological wellbeing variables explained an additional 15% of the variance in children’s adjustment and the omnibus test for the second step was also significant: *F*(8, 86) = 4.14, *p* < .001. After accounting for the effects of perceived financial difficulties and child’s gender, maternal parenting stress was significantly related to children’s adjustment problems. The interaction terms explained an additional 4% of variance in child adjustment difficulties and the omnibus test for the third step was significant: *F*(13, 81) = 2.87, *p* = .002. None of the interaction terms was a significant predictor of child adjustment difficulties showing that there were no differences between family types in the relations between the parenting and maternal wellbeing variables and child adjustment.[Table-anchor tbl3]

## Discussion

The children of single mothers by choice in the present study were found to experience similar levels of parenting quality to the comparison group of children in traditional two-parent families. For positive aspects of parenting as assessed by interview, there was no difference between the two family types, with mothers and their children in both the solo mother and two-parent families showing high levels warmth and interaction. Neither was there a difference in the quality of mother–child interaction as assessed through observation. There was a difference, however, for negative aspects of parenting as assessed by interview, with a lower frequency of conflict between mothers and their children in solo mother than in two-parent families. This finding remained when the analysis was repeated without covariates, suggesting that this represents a genuine difference between the two family types. Whereas this may seem to be an anomalous finding, in a previous study of a different sample, solo mothers with 2-year-old donor-conceived children were rated as feeling less anger toward their children than were a comparison group of partnered mothers and the children showed fewer emotional and behavioral problems (Murray & Golombok, 2005b). Although divorced and unmarried single mothers have been shown to experience raised levels of psychological problems, this was not found to be the case in the present study of single mothers by choice. The solo mothers did not differ from the partnered mothers in terms of anxiety, depression, or stress associated with parenting.

With respect to the psychological wellbeing of the children, no differences were found between the children in solo mother and two-parent families for emotional and behavioral problems as assessed by the Strengths and Difficulties Questionnaire completed either by mothers or the children’s teachers. In addition, the assessment of the presence of psychiatric disorder by a child psychiatrist showed no difference between family types in the proportion of children rated as having a psychiatric disorder. As the child psychiatrist was unaware of the child’s family type, these findings provide important validation for the mother’s reports. It is important to note that almost two thirds (64%) of the children with a slight or marked psychiatric disorder had a developmental disorder which is unlikely to be related to family type. When the four children with a marked psychiatric disorder were removed from the sample, the findings did not change for any of the parenting or child adjustment variables.

Although the children of solo mothers were no more likely to experience psychological problems than were those with a mother and father, higher levels of financial difficulties, and higher levels of parenting stress were each associated with higher levels of children’s emotional and behavioral problems within the solo mother families. As discussed above, financial hardship is one of the key predictors of psychological problems in the children of divorced or unmarried single mothers (Hetherington & Stanley-Hagan, 2002; McLanahan & Sandefur, 1994; Pryor & Rodgers, 2001; Amato, 2000, 2005), and it seems from the present study that financial hardship also has a negative impact on children’s psychological wellbeing in families where the mother has made an active decision to parent alone. The finding that stress associated with parenting was associated with child adjustment problems in the solo mother families is consistent not only with previous research on single mothers by choice (Chan et al., 1998) but also with the broader research literature on divorced and unmarried single-mother families (Amato, 2000, 2005; Dunn et al., 1998; Hetherington & Stanley-Hagan, 1999; Pryor & Rodgers, 2001). It is important to emphasize, however, that the solo mother families did not differ from the two-parent families in the association between financial hardship, parenting stress, and child adjustment problems, indicating that these risk factors were operating in a similar fashion in both family types.

A potential risk factor for the children of single mothers by choice that does not exist for children from other types of single-mother family is their donor conception. As a result, the children grow up unaware of the identity of their biological father. The low level of psychological problems among the children of single mothers by choice in the present study suggests that lack of knowledge of the identity of their biological father does not have a negative impact on their psychological wellbeing. However, the mean age of the children was only 5-1/2 years at the time of study. Research on adoption has found that adopted children show an increased interest in their biological parents at adolescence, the time at which issues relating to identity become salient (Brodzinsky, 2011; Grotevant & Von Korff, 2011). This suggests that the children of single mothers by choice may similarly become more interested in their biological father at adolescence and the absence of information about his identity may produce challenges at that time. In a qualitative study of solo mothers with 3–11-year-old donor-conceived children in Israel, Weissenberg and Landau (2012) reported that all of the children expressed a wish for a father.

A potential difficulty with the study is that differences between the solo mother and two-parent families may not have been identified due to the modest sample sizes. However, it would have been possible to detect a *d* (standardized difference between means) as small as 0.27 as statistically significant, for a power of 0.80. Thus, to the extent that significant differences between family types may not have been detected due to insufficient power, these differences would have been small. For the regression analysis which involved 103 families, 2 predictors in step one and 13 predictors in Step 3, it was possible to detect effects sizes as small as 0.20 as significant, for a power of 0.80. Indeed, an effect size of 0.21 was found to be significant. Although larger samples would have been desirable, this is the first controlled, in-depth study to focus on school-age children born to heterosexual single mothers by choice and thus sheds light on the functioning of this new family form.

A further limitation of the study was that not all of the parenting variables derived from the interview showed interrater agreement of 80% or above which could also have resulted in the failure to identify significant effects. However, the coding of the interview variables that did not reach this threshold (two variables included in the positive parenting factor) involved the use of nonverbal cues such as facial expression and gestures that were not available to the second rater. Thus the interrater reliabilities of these interview variables may be underestimates. When the positive parenting factor was reanalyzed with these two variables removed, the finding did not change. The mutuality variables showed a restriction in the range of scores rather than poor interrater agreement and have been shown to be reliable in studies of more diverse samples (Deater-Deckard & Petrill, 2004), including studies by our own research group (Ensor & Hughes, 2010; Golombok et al., 2011).

For reasons of confidentiality, selection of the families was carried out by the clinic rather than the researchers and it was not possible to match the two groups on variables other than the age and gender of the child. As solo mothers tend to be older than partnered mothers when they embark upon donor insemination and have fewer children, the samples reflected these demographic differences. When these variables were found to correlate with a dependent variable they were entered into the analysis as covariates. Although only 57% of the teachers completed the SDQ, the teachers’ scores were highly correlated with the mothers’ scores. In fact, the correlation of 0.53 between the mothers’ and teachers’ scores was higher than is usually found between mothers’ and teachers’ ratings of child adjustment problems (Zaslow et al., 2006). Moreover, there was no difference in the proportion of missing teachers’ questionnaires between family types and no difference in mothers’ SDQ scores between families with and without teacher questionnaires. Thus, there did not appear to be a bias toward higher levels of adjustment among the children whose teachers completed questionnaires. One reason for the lower number of teachers’ than mothers’ questionnaires was that 15 of the mothers (seven solo and eight partnered) did not wish their child’s teacher to be asked to participate in the study in order not to draw attention to their child. When these families were removed from the calculation, the response rate from teachers approached 70%.

A particular advantage of the study was that the children in the comparison group of two-parent families had also been conceived by donor insemination thus controlling for the use of donor conception by the single mothers by choice. A further advantage of the study was the multimethod (interview, observation, and questionnaire), multi-informant (mother, child, and teacher) design as single mothers by choice may play down difficulties and tend to present their families in a favorable light due to the negative attitudes they experience from others and because of their own concerns about providing a positive family environment for their children. The observational measure is especially useful in this regard as it is difficult to “fake good” with observational measures (Kerig, 2001). A further benefit of the observational measure is that it provided an assessment of the quality of dynamic interactions between parents and their children that cannot be captured by interview or self-report (Aspland & Gardner, 2003; Bakeman & Gottman, 1997; Funamoto & Rinaldi, 2015; Hartmann & Wood, 1990).

Research on solo mother families is of theoretical interest as it provides an opportunity to examine the impact of single motherhood on children’s wellbeing in the absence of the risk factors such as parental conflict, economic hardship, and maternal mental health problems that are associated with psychological problems in the children of divorced single mothers and unmarried single mothers whose pregnancies were unplanned (Golombok, 2015). The finding that the children of solo mothers showed positive psychological functioning and did not differ from their counterparts in two-parent families suggests that single motherhood, in itself, does not have negative psychological consequences for children. Interestingly, when the group comparisons of parenting and child adjustment were conducted without covariates, the findings were identical, indicating that differences between solo mother and two-parent families were not being masked by the inclusion of covariates in the analyses. The fact that the solo mothers made an active decision to parent alone rather than finding themselves in this situation unintentionally may have contributed to the positive outcomes for these families; children born by donor insemination to single mothers by choice are extremely wanted children whose mothers went to great lengths to conceive them whereas divorced single mothers and unmarried single mothers who had unplanned pregnancies did not set out to parent alone. Thus, it is conceivable that the intention to be a single parent contributes to positive mother–child relationships and, consequently, to positive child outcomes. In contrast, more negative mother–child relationships and child outcomes may result from parenting alone when single motherhood had not been planned or desired. Although it is not known why there was a lower frequency of mother–child conflict in solo mother than in two-parent families, it may be relevant that, unlike the mothers from two-parent families, the solo mothers did not have to cope with the potentially stressful experience of their partner’s infertility and his lack of a genetic relationship with the child.

The finding that perceived financial difficulties and parenting stress were associated with increased levels of psychological problems in children in both family types is in line with the prediction derived from developmental contextual systems theory (Overton, 2014, 2015) that children’s adjustment difficulties would not be a function of single motherhood but instead would be associated with financial hardship and maternal mental health problems. The findings of the present study of solo mother families add to the growing body of evidence from studies of other types of single-mother family (Chan et al., 1998; Demo & Acock, 1996; Demuth & Brown, 2004; Dunn et al., 1998; Kiernan & Mensah, 2010; Lansford, Ceballo, Abbey, & Stewart, 2001), as well as the literature on new family forms more generally (e.g., Bos & Gartrell, 2010; Bos, Gartrell, Peyser, & van Balen, 2008; Farr, Forssell, & Patterson, 2010a, b; Golombok, 2015; Golombok, Blake, Casey, Roman, & Jadva, 2013; Golombok et al., 2014), showing the relative importance of family processes over family structure for children’s psychological adjustment.

## Figures and Tables

**Table 1 tbl1:** Means, Standard Deviation, F, and p Values for Sociodemographic Information by Family Type

Demographic	Solo mothers		Partnered mothers		*F*	*p*
	*M*	*SD*	*M*	*SD*		
Age of mother	44.08	3.90	39.21	4.30	36.05	<.001
Age of child	68.17	19.96	63.30	16.78	1.79	*ns*
	*N*	%	*N*	%	χ^2^	*p*
Child gender						
Male	26	51%	31	60%	.77	*ns*
Female	25	49%	21	40%		
Siblings						
None	31	61%	16	31%	9.39	.009
One	15	29%	26	50%		
Two or more	5	10%	10	19%		
Mother’s working status						
Not working	14	28%	14	27%	8.54	.014
Part-time	16	31%	29	56%		
Full-time	21	41%	9	17%		
Perceived financial difficulties						
None	40	78%	43	83%	3.19	*ns*
Minor	7	14%	6	11%		
Definite	4	8%	3	6%		
Mother’s education						
Below university degree	16	32%	24	46%	2.90	*ns*
Undergraduate degree	18	35%	17	33%		
Postgraduate degree	17	33%	11	21%		
Mother’s psychiatric contact						
None	45	88%	42	81%	3.08	*ns*
General practitioner	6	12%	7	13%		
Outpatient	0	0%	3	6%		

**Table 2 tbl2:** Means, Standard Deviation, F and p Values for Positive Parenting, Negative Parenting, Mothers’ Psychological Well-Being, and Mutuality by Family Type

Parenting	Solo mothers		Partnered mothers		*F*	*p*
	*M*	*SD*	*M*	*SD*		
Positive parenting						
Expressed warmth	4.14	.83	4.00	.74	1.00	ns
Mother-to-child warmth	2.76	.43	2.65	.48	.31	ns
Child-to-mother warmth	2.68	.51	2.62	.56	.33	ns
Mother’s enjoyment of play	3.38	.60	3.38	.74	1.42	ns
Amount of interaction	2.56	.50	2.54	.57	1.83	ns
Quality of interaction	3.14	.63	3.15	.63	.04	ns
Negative parenting						
Conflict	1.08	.59	1.04	.65	1.03	ns
Frequency of battles	5.24	2.21	6.17	1.38	12.91	.001
Level of battles	1.41	.72	1.37	.52	.52	ns
Criticism	.73	.75	.65	.78	.13	ns
Psychological well-being						
Trait Anxiety Inventory	35.62	7.64	37.33	9.56	1.22	ns
Edinburgh Depression Scale	5.60	3.62	5.64	4.09	.38	ns
Parenting Stress Index	62.86	13.33	63.49	14.88	.24	ns
Mutuality						
Child responsiveness	5.42	.72	5.42	.69	.75	ns
Mother responsiveness	5.56	.89	5.88	.49	1.40	ns
Dyadic reciprocity	2.76	.98	2.35	.94	2.22	ns
Dyadic cooperation	2.93	1.49	2.86	1.30	.86	ns

**Table 3 tbl3:** The Relationship Between Demographic Characteristics, Parenting, Maternal Wellbeing, and Child Adjustment Difficulties

	Child adjustment difficulties			
Step	Variable	B	*SE* B	b
Step 1	Perceived financial difficulties	2.19	.74	.29*
	Child’s gender	−2.24	.89	−.25*
Step 2	Perceived financial difficulties	1.57	.74	.21*
	Child’s gender	−2.02	.86	−.22*
	Family type	.24	.42	.05
	Positive parenting	.81	.45	.18
	Negative parenting	.30	.45	.07
	Maternal depression	−.07	.15	−.06
	Maternal anxiety	.03	.07	.06
	Maternal parenting stress	.11	.04	.34*
Step 3	Perceived financial difficulties	1.40	.75	.19
	Child gender	−2.10	.87	−.23*
	Family type	.23	.42	.05
	Positive parenting	.93	.48	.21
	Negative parenting	.64	.49	.15
	Maternal depression	−.10	.15	−.08
	Maternal anxiety	.05	.07	.09
	Maternal parenting stress	.09	.04	.30*
	Family Type × Positive Parenting	.76	.48	.17
	Family Type × Negative Parenting	.64	.48	.15
	Family Type × Maternal Depression	−.11	.15	−.09
	Family Type × Maternal Anxiety	.10	.07	.19
	Family Type × Maternal Parenting Stress	−.04	.04	−.12
DV	Child adjustment difficulties	*R*^2^ = .13 for Step1		
		D*R*^2^ = .15 for Step 2		
		D*R*^2^ = .04 for Step 3		
*Note*. DV = dependent variable.				
* *p <* .05.				
